# A community’s experience during and after the Ebola epidemic of 2014—2016 in Sierra Leone: A qualitative study

**DOI:** 10.1371/journal.pntd.0009203

**Published:** 2021-02-25

**Authors:** Rianna T. Murray, Laura Briggs Drew, Christina Memmott, Ya-Maila Bangura, Elisabeth F. Maring

**Affiliations:** 1 Maryland Institute for Applied Environmental Health, University of Maryland School of Public Health, College Park, Maryland, United States of America; 2 Department of Family Science, University of Maryland School of Public Health, College Park, Maryland, United States of America; 3 Public Health Science Program, University of Maryland School of Public Health, College Park, Maryland, United States of America; 4 Department of Government & Politics, University of Maryland College of Behavioral and Social Sciences, College Park, Maryland, United States of America; University of Texas Medical Branch, UNITED STATES

## Abstract

**Background:**

The 2014–2016 Ebola epidemic devastated families and communities throughout West Africa. Due to its high mortality rate and infectious nature, most Ebola research to date has focused on healthcare response and interventions; however, little is known about the experiences of Ebola survivors and communities. This qualitative study aimed to better understand the lived experiences of community members, including children, during and after the Ebola epidemic in Sierra Leone.

**Methods:**

During June 2016 and June 2017, we conducted four focus groups comprised of primary school students, female caretakers, male caretakers, and teachers, and two individual in-depth interviews with local nurses in Calaba Town, a small village outside of Freetown. Interviews were recorded, transcribed verbatim, and coded using a modified grounded theory methodology.

**Findings:**

All participants shared that they experienced significant challenges during and after the Ebola epidemic. During the epidemic, participants endured daily life challenges pertaining to fear, financial distress, and school closures. They also experienced suffering, loss, isolation, grief, and compromised culture. Confusion and distrust were also prevalent during the epidemic, with participants reporting confusion around Ebola transmission and distrust in the government and healthcare services. We also found that the struggle for food and grief stemming from the loss of loved ones continued more than a year after the epidemic ended. Despite Sierra Leone being declared Ebola-free, stigma and fear persisted and community members shared their continuing distrust of the government due to their actions during and after the epidemic.

**Conclusions:**

The findings of this qualitative study reveal that the Ebola epidemic was a traumatizing period for the Calaba Town community, and that confusion and distrust toward the government health care system have continued. Future studies should explore the extended impact of the epidemic on communities, including long-term psychological, social, and economic consequences of this outbreak.

## Author summary

The Ebola epidemic of 2014–2016 in West Africa was the largest in history, resulting in over 28,000 infections and more than 11,300 deaths across Sierra Leone, Liberia, and Guinea. Little is known about the lived experiences of communities, families, and children during and after the epidemic, and this qualitative study sought to better understand these experiences in a community in Sierra Leone. Through focus groups and individual interviews, we discovered that participants endured daily life challenges pertaining to fear, financial distress, and school closures. They also experienced suffering, loss, isolation, grief, and compromised culture, especially regarding funeral traditions. Children expressed being unhappy while schools were closed due to their inability to learn and interact with their classmates. Healthcare workers reported the stigmatization that they experienced, along with fear of an unfamiliar disease that led to the deaths of their colleagues. Distrust in the government, particularly due to their epidemic response, fueled feelings of confusion about Ebola transmission and treatment, and an overall lack of trust in healthcare services. Despite Sierra Leone being declared Ebola-free in March 2016, stigma and fear persisted and community members shared their continuing distrust of the government.

## Introduction

An outbreak of Ebola emerged in Guinea in early 2014 and crossed geographical boundaries, affecting other African countries including Sierra Leone, Liberia, Nigeria, and Mali. The outbreak was unprecedented and it jolted West Africa between 2014–2016, with over 28,000 Ebola cases reported [1]. It became the largest Ebola outbreak in history, claiming more than 11,300 lives before it was contained in March 2016 [1]. There were a total of 14,124 cases of Ebola in Sierra Leone, with 3,956 of these resulting in deaths [1]. The Ebola epidemic was a complex public health crisis with various sociological, ecological, and environmental drivers, including a poor healthcare infrastructure.

The incidence rate of Ebola Virus Disease (EVD) in the Western Area (WA) of Sierra Leone, which includes our study area of Calaba Town, was approximately 17.32–36.10 per 10,000 people between 2014 and 2015 [2]. The WA Region, which contains two of the country’s 14 districts, had more than half of the reported cases and deaths [2]. Sierra Leone faced unique challenges during this epidemic that were influenced by the country’s recent civil war (1991–2002), including a frail healthcare infrastructure. Sierra Leone lost $1.4 billion in GDP in 2015 as a result of the Ebola epidemic [3]. The country’s already weakened and overburdened healthcare system contributed to the widespread devastation caused by Ebola in Sierra Leone [4,5].

In addition to healthcare infrastructure, trust is one of the most important factors in the success of a healthcare system [6]. Trust is conceptualized as the expectation that healthcare providers will demonstrate knowledge, skill and competence, and act with integrity in the patient’s best interest, leading to overall social wellbeing [7,8]. Trust has been described as the ultimate test of success or failure of a healthcare system [6], and a lack of trust in public health organizations can undermine public confidence in their leadership and policy [9]. Declining trust in the national healthcare system was identified as a potential factor in the reluctance of citizens in both Sierra Leone and Liberia to seek medical care during the Ebola epidemic [10–12]. Distrust of the government and the public health service can therefore present dangerous complications during an infectious disease outbreak.

The Ebola epidemic also highlighted global disparities in health care resources and the dire need to improve the healthcare system in Sierra Leone. International efforts from several aid agencies focused on identifying Ebola cases, providing treatment, and preventing the transmission of Ebola. However, since the end of the epidemic in early 2016, little research has focused on the experiences of communities during and after this crisis. Additionally, the specific experiences of children have not been assessed. Our study seeks to address this gap by exploring the lived experiences of members of a semi-rural community in Sierra Leone during and after the Ebola epidemic. We focused on understanding the community members’ perceptions, attitudes and beliefs about Ebola and the ways in which daily life was altered during the epidemic, including the phenomenon of living under the constant threat of contracting Ebola and returning to life after the epidemic. We interviewed community members who lived through the epidemic and experienced Ebola in their families and community. However, none of the individuals who we interviewed were survivors of the disease.

We also aimed to obtain an understanding of the community’s trust in the healthcare system and government response to the epidemic. We did not have *a priori* hypotheses, but aimed to uncover the range of experiences of this community. Through a series of semi-structured open-ended interviews with community members, healthcare professionals, and students from a local school, we sought to understand the lived experience of the Calaba Town community, a semi-rural community outside of Freetown, during and after the Ebola epidemic in Sierra Leone. Given that the 2020 COVID-19 global pandemic exposed similar feelings of confusion and distrust in many populations across the world [13–16], qualitative studies like ours are important to better understand the origins of confusion and distrust in public health messaging during an infectious disease outbreak as well as the lived experiences of community members during such times.

## Methods

### Ethics statement

Ethics approval was granted by the Institutional Review Board (IRB) of the University of Maryland (UMD). All study participants agreed to participate in the study on a voluntary basis after verbally providing informed consent (adults) and informed assent (children). The UMD IRB reviewed and approved the verbal assent protocol for children and verbal consent protocol for adults. The consent and assent processes were explained to participants by trained study staff using the IRB-approved consent and assent forms. The UMD IRB waived the need for parental consent of child participants since children’s risk of participating in the study was low, and to ensure anonymity of participants. No formal ethics review board was present in or near Calaba Town. However, prior to implementation, we discussed the qualitative study with leaders at the school, including the Vice Principal, who is also a leader in the community. They encouraged us to implement our qualitative study as it would provide an opportunity for members of the community to share their experiences during the Ebola epidemic with others, which the community members also reiterated.

### Study Overview

We conducted a qualitative study with individuals from Calaba Town, a small village with limited access to resources approximately 20 km southeast of Freetown, the capital of Sierra Leone. This community was selected for our study because of its unique location, which makes it neither rural, like towns in the Eastern provinces, nor urban, like Freetown. At the time of study implementation, little was known about how experiences during the Ebola outbreak differed between urban and rural locations. However, we hypothesized these disparities existed and that interviewing members of the Calaba Town community would provide unique perspectives about life during and after the outbreak. Additionally, our ongoing relationship with the Calaba Town community and a non-government primary school in the area through Public Health Beyond Borders (PHBB), a student-led organization at the University of Maryland, influenced our choice of Calaba Town as the study site. PHBB previously conducted community-based research in this location, including health needs assessments and educational workshops on health and hygiene with children and adults in this community, and in this study, we aimed to better understand the community’s experiences during and immediately after the Ebola epidemic.

### Data collection

We chose participants via purposive sampling. Eligibility criteria included residing in the Calaba Town community during the 2014–2016 Ebola epidemic and willingness to have their interview recorded. To ensure participants felt comfortable during the interview, we conducted interviews at a local school and church, whichever was more convenient for the participants. We estimated that a sample size of approximately 20 individuals was necessary to reach data saturation.

In June of 2016 and 2017, trained student interviewers conducted open-ended interviews with participants, which were designed to take 45–60 minutes to complete. Basic demographic information was collected at the beginning of the interview. All interviews were conducted in English and a community member who is fluent in English and Krio (second language commonly spoken in Sierra Leone), assisted with Krio translations when necessary. Our interview guide focused on two major domains: 1) Experiences during the Ebola epidemic, and 2) Changes at the community level after the Ebola epidemic.

We conducted four focus groups (primary school students, female caretakers, male caretakers, and primary school teachers) and two individual interviews with nurses who worked during the Ebola epidemic. In 2016, four boys and four girls, who were in class six (the terminal primary school class) and between the ages of 12 and 14, participated in the student focus group. That same year, we conducted a focus group with five women (three stonebreakers, one petty trader, and one home health educator). All women had children or were guardians for orphans at the school. In 2017, three men (two construction workers and one Non-Governmental Organization [NGO] employee) whose children had attended or were currently attending the school participated in the male caretakers focus group. Additionally, we conducted a focus group in 2017 with eight primary school teachers. We also conducted two individual interviews with nurses who had worked during the Ebola epidemic (one interview conducted each year). During the interviews, we provided snacks and beverages and participants received hygiene products for their participation.

### Analysis

Interviews were audio-recorded and transcribed verbatim into Microsoft Word by students at the University of Maryland, who received training in qualitative research methods. Four researchers then independently hand-coded each of the interviews in Microsoft Word to identify major themes using modified grounded theory methodology, which informed our research approach. Grounded theory methodology is a qualitative approach for developing theory that is grounded in the data which is then systematically gathered and analyzed [17]. In grounded theory methodology, the data can generate a theory, or existing theories can be elaborated or refined as additional data are compared against them [17]. In this methodological approach, a story about the people, social processes, and situations is composed by the researchers via the data that is shared in the interview process [18]. The overarching hypothesis of our study was that the Ebola epidemic was a catastrophic event that changed peoples’ daily lives in ways not previously experienced or explored. Our qualitative approach was also influenced by phenomenology, as we aimed to explore the participants’ lived experiences and create meaningful descriptions of these events [19,20].

To inform the grounded theory process, the four researchers met and compared the codes that were similar and unique across each of the interview transcriptions. This allowed the researchers to identify which codes were most common and codes that were unique. Codes were then grouped together into categories to inform the development and establishment of themes and sub-themes. This brainstorming session, which included the development of an extensive concept map, elucidated the core meaning of the data. Prior to the 2017 interviews and focus groups, we made slight modifications to the interview guide since themes and ideas requiring further investigation had been revealed in the 2016 interview and focus groups. To ensure data validity between investigators, members of the research team conducted data triangulation by comparing the coding of the data. These comparisons regularly supported data validity between investigators. This collaborative process of confirmation and modification helped ensure that the emerging framework was grounded in the data [21].

## Results

### Participant demographics

In total, 19 adults, between 23 and 53 years of age, participated in focus groups and individual interviews. The majority of the adult participants were female (67%) with varying levels of education (Table 1). Participant occupations included nine school personnel, two nurses, two construction workers, two stone breakers, one NGO employee, and one petty trader. One participant was unemployed at the time of the interview; however, she previously worked as a home health educator. One participant’s occupation was unknown. Marital status differed among participants, with 32% of participants reporting that they were married, 32% were single, 11% were widowed, and 26% were of unknown marital status. Adult participants had varying numbers of children, from no children (17%) to six children (5.5%). Number of people living in their homes also varied, from two people (5.5%) to nine people (5.5%). The eight students in grade six who participated in the focus group (four boys and four girls), were between the ages of 12 and 14. Their parents’ occupations varied; however, 50% of their mothers were petty traders, 37.5% of their fathers were stonebreakers, and one student’s father was deceased.

**Table 1:** Characteristics of study participants.

Participants	n (%)
**Adults (n = 19)** Gender	
Male	7 (37%)
Female	12 (63%)
Age	
20–29	4 (21%)
30–39	3 (16%)
40–49	5 (26%)
≥ 50	2 (11%)
Unknown	5 (26%)
Education	
Never attended school	1 (5%)
Finished primary school	2 (11%)
Teacher’s Certificate	8 (39%)
Accounting Diploma	1 (5%)
Nursing Degree	2 (11%)
Unknown	5 (26%)
Occupation	
Nurse	2 (11%)
School Personnel	9 (47%)
Construction	2 (11%)
NGO employee	1 (5%)
Stone breaker*	2 (11%)
Petty trader**	1 (5%)
Unemployed***	1 (5%)
Unknown	1 (5%)
Relationship status	
Married	6 (32%)
Single	6 (32%)
Widowed	2 (11%)
Unknown	1 (5%)
Number of living children	
0	3 (16%)
1	3 (16%)
2	3 (16%)
3 or more	9 (47%)
Unknown	1 (5%)
**Children (n = 8)**	
Gender	
Male	4 (50%)
Female	4 (50%)
Age	
12	3 (37.5%)
13	4 (50%)
14	1 (12.5%)
Mother’s Occupation	
Petty trader	4 (50%)
Teacher	1 (12.5%)
Stone breaker	2 (37.5%)
Father’s Occupation	
Petty trader	1 (12.5%)
Teacher	2 (25%)
Stone breaker	3 (37.5%)
Police officer	1 (12.5%)
Deceased	1 (12.5%)

* Stone breaker–this job entails manually breaking large stones into smaller gravel for construction projects.

** Petty trader–this job entails buying and selling consumer goods, such as food and household items, on a small scale. Petty traders do not have an official place of business and typically walk around the village with their goods on their person.

*** Participant was formerly employed before the epidemic as home health educator.

Within the domains of 1) Experiences during the Ebola epidemic, and 2) Changes at the community level after Ebola epidemic, four themes emerged from our interviews and focus groups: the proliferation of daily life challenges, feelings of suffering and loss, concepts of confusion and distrust, and the challenge of moving forward after the end of the epidemic (Table 2). Several sub-themes also emerged during data analysis, revealing additional experiences during and after the Ebola epidemic (Table 2).

**Table 2:** Themes and sub-themes from focus groups and interviews with members of the Calaba Town community after the Ebola epidemic.

Domains	Themes	Sub-themes
Experiences during the Ebola epidemic	Daily life challenges	Change in family dynamics/relationships
		School closures
		Fear
		Financial stress
	Suffering and Loss	Isolation
		Idle time
		Hunger
		Disparities in suffering
		Compromised culture
		Managing grief
	Confusion and distrust	Belief that Ebola was man-made
		Misunderstanding Ebola transmission
		Fear of government services and healthcare
		Trust of NGOs more than government
Changes at the community level after Ebola epidemic	Moving forward after the epidemic	Ongoing stigma and fear Impact of losing loved ones Financial challenges Caring for displaced family members Continuing distrust of government

### Daily life challenges during Ebola

For all of our participants, daily life challenges were a struggle during the Ebola epidemic. Within this theme, participants shared the changes in family dynamics and relationships that they endured, the impact of school closures, and the overwhelming feelings of fear, which permeated their community. Lockdowns and the inability to work also led to extreme financial stress for families.

#### Change in family dynamics/relationships

Relationships were strained because husbands and wives were unable to be intimate with one another when one was sick during the Ebola epidemic. Additionally, due to fear of contracting their partner’s illness, wives were unable to care for their sick husbands. For many women, this was difficult because they believed their roles and responsibilities as wives had been compromised.

"Well, I suffered a lot. My husband was also seriously sick during the Ebola, and I could not touch him. I was forced not to care for him, and sometimes we don’t sleep in the same place even as husband and wife, so I have to stay away from him. It was very bad." - 35 year old, female, Stonebreaker

#### School closures

During the Ebola epidemic, the government of Sierra Leone closed the schools for six months. Many mothers expressed that it was challenging for them to manage their usual daily tasks while having their children home during the day. Their children were bored, unhappy to be out of school, and they wanted to play with their friends. They were also hungry, which was stressful because food access was limited during the height of Ebola. Overall, children shared that they missed their classmates and attending school. A 13 year old grade six student shared, *"I was unable to go to school; I was unable to play with my friends*. *I feel bad*.*"* One of her classmates, another 13 year old grade six student, similarly described the experience as, *“Yes*, *we are indoors*, *and I take my book to study*. *I feel bad because I do not go to school*.*”* All of the students shared that being unable to attend school was a challenging experience, especially for those whose national exam preparations were put on hold due to school closures.

The government aired school programs over the radio to help students continue their education while schools were closed; however, not all children benefited from the program. A 32 year old male teacher explained, “*Well*, *the problem there is that not everyone can access the radio station to listen*. *Maybe it’ll be there but they cannot afford to get a battery*.*”* While not all children were able to benefit from the radio program, the community still acknowledged the existence of the government’s effort.

#### Fear

Living with fear, tension and anxiety was common during the epidemic, and participants reported a constant fear of someone dying due to Ebola. Fear of the disease resulted in changes to social and cultural norms and to the development of antisocial behaviors, especially before it was known how the disease was spread.

“Ebola-it was terrible, as I said. It ravaged the whole country. A lot of nurses died, doctors died, lay people died. And because of that, when it was here, we were like afraid of each other. Even in church we are sitting three per bench, nobody was touching anybody. Even at home, there was not that cohesiveness.”– 53 year old, female, Nurse

Healthcare professionals were ostracized from their own communities by their family, friends and neighbors due to fear that they would transmit Ebola to others. Ebola undermined the trust that people had for each other, healthcare workers, and for health facilities because of the consistent fear of contracting the disease.

“Especially for us as health workers people were shying away from us because they said all of us have Ebola. When going out, it was during this epidemic that nurses stop wearing the uniform. Normally we used to dress fully when going to work, but when Ebola break out if you wear the uniform and went into a cab, you would be dropped out of that cab. People were shying away from us because they said we are the ones that brought in Ebola so we are not welcome.”– 53 year old, female, Nurse

One healthcare worker even described being displaced from her home during the epidemic. She was stigmatized by neighbors because of her profession, and was forced to leave the home she shared with her parents and her son. Instead, she rented a room in an apartment where she kept her job a secret from her roommates.

“Because when I go to work, I decide to leave the place at that time because they told the landlord that I was going to work and I want to kill everybody. So I decide I don’t want my mother and dad to get confused, so I lived in another place for about two to three months, so after which I come back home, so that was the situation. It’s not easy. People are stigmatized because if you are a nurse. Even if you wear your uniform, during that time, if they know they will not pick you up. If they know you are a nurse, they will not pick you up.”– 29 year old, female, Nurse

Later in the interview, she also mentioned the following:

“I secure a room where I stayed. No one knew I’m a nurse; in the morning I just hang my bag, go after work.”– 29 year old, female, Nurse

One of the participants of the men’s focus group worked for an NGO and was responsible for discarding and replacing the belongings of Ebola victims. He began his duties after undergoing a three day training and expressed concern about the danger of his tasks with so little training:

“It was difficult because I am not a trained nurse. I am not a trained doctor. We were only trained for about three days and we were sent to the field. It was dangerous. It was tedious. It was hard for us, but we managed to get through it.”– 47 year old, male, NGO worker

People became afraid of each other and neighbors no longer welcomed each other, with one participant remarking *"It was like we see ourselves as enemies*, *you know*? *We are afraid of each other*. *We cannot touch hands*.*"*– *33 year old*, *female*, *currently unemployed*, *previously a Home Health Educator*

This disintegration of previously close-knit communities was a difficult social consequence to endure since this was not the typical way of life in Sierra Leone.

“… in our setting we used to shake hands, but during Ebola we could not. We are used to hug each other, you know, that one too. It didn’t happen. But it was difficult because it has been in our culture for ages! So for just to shut all these things out- it was not easy for people.”– 53 year old, female, Nurse

Not only were community members mistrustful of each other, but community members who typically helped each other were afraid to do so during the epidemic, feeling that they had no other choice in order to protect themselves.

“We have one kid who is at Allentown when we went to the house, they told us that the dad died and the mother. The child was there. He cried until he died … No one touched him. He cried until he died … We are afraid. If anyone see you going to that particular houses, they will disown you. Not only stigmatize, they will disown you.”– 29 year old, female, Nurse

Fear of contracting the disease was even prominent within families, causing family members to be wary of each other. One teacher noted:

“You are even afraid of your closest relative. As soon as they say, ‘I’m sick’, you’ll be afraid of your relative and even your child.”–>50 year old, male, Teacher

#### Financial stress

Financial stress became a prominent burden during the Ebola epidemic. Inability to work fueled a cycle of constant worrying about money and being able to provide for their families.

“Everything was difficult. You cannot move, and if you cannot move, you cannot work. And if you don’t work, you don’t have money. And if you don’t have money, you cannot buy food.”–>50 year old, male, Teacher

Having enough food and fighting hunger was a daily struggle. Even neighbors who would typically assist each other in time of need could not do so during the epidemic for fear of not having enough for themselves and their own families.

“We don’t have enough money to buy, neither enough food to eat. And we don’t even have a place to go and buy because we can’t go out. And when you go to somebody’s home, they say ‘No you have to stay at home.’ It is true. Unless when somebody is asking food from you, well either sell or give for free. If there is any, if they are having enough.”– 35 year old, female, Stonebreaker

Some self-employed community members were able to continue working, but there was no one to buy their goods.

“Well sometimes you don’t have money, but you cannot get this food stuff. Other times there is no money, you don’t have money, and you don’t have a way to go out to get money. Even if you broke your stones, who is coming to buy them? Nobody. So you are stuck with no one to buy them. And so, you have to suffer.”– 47 year old, female, Stonebreaker

### Suffering and loss

To better understand the impact of Ebola on the community, we asked participants about their lived experiences during the Ebola epidemic. Within the theme of suffering and loss, the following sub-themes emerged from the interviews: isolation, idle time, hunger, disparities in suffering, compromised culture, and managing grief.

#### Isolation

Participants across all focus groups shared their feelings of being isolated during the Ebola epidemic, as community members were unable to leave their homes and could not visit their friends, family members, or neighbors, whether they were healthy or sick. Church services were also suspended, and people were unable to pay their respects to loved ones. In addition to not being able to leave their homes and attend activities in their community, participants shared that they could not welcome people in their homes due to fear of contracting Ebola.

“During the Ebola, you don’t allow strangers. You don’t allow strangers. Because you don’t know, maybe if they know that this home is being quarantined, they will leave their home and go elsewhere. So when you go to maybe let them in, you become infected. So during Ebola, no visitor is allowed at your home.”– 29 year old, female, Nurse

Participants also shared that they isolated themselves when they were sick because they did not want their neighbors to report their illness to the authorities. Every illness was treated as a potential Ebola case, resulting in a widespread fear of being taken away from their homes and never seeing their loved ones again. The Nursery School teacher described her experience in the following quote:

“Well that what’s happened to me. During the closing, I was not well. I decided to stay in my room and not even come outside …because during that time, if they hear that you are sick, you will go out and you will not come again. That’s why I decided to stay in. I was suffering, I was having ulcer.”– 42 year old, female Teacher

#### Idle time

In addition to not being able to freely leave their homes, participants shared that their daily activities were restricted. Because they were unable to work and children could not attend school, there was little they could do during that time. For participants who were not part of the healthcare system, there was nothing they could do to help pass the time.

"During Ebola, no work. We do not do anything. We do not even allow our children to go far from the home. While sometimes we are to go and sleep, even though we are suffering, but we have no alternative but to stay at home."– 37 year old, female, Petty Trader

#### Hunger

Because individuals were unable to work, their families suffered financially during the Ebola epidemic. This made it difficult for families to purchase necessities, including food and coal, to prepare meals for their families. Because of the financial strain families experienced, extreme hunger was common during the Ebola epidemic. During the school closures, children were restricted to staying in their homes and they were bored and hungry, which was particularly difficult for their parents to manage.

"At that time, it was very hard to for us to get food, so that was very difficult for us. We really suffered a lot at that time because some people actually lived day by day except to go out and fight for food. That was really stressful for us.”– 28 year old, male, Teacher

#### Disparities in suffering

During the Ebola epidemic, some groups and communities were affected more than others. Participants shared their perspectives that women were more burdened during the epidemic because of their role as caregivers of the household and having to care for sick family members. Participants perceived that women were more likely to die during Ebola and pregnant women were particularly vulnerable due to limited access to healthcare. A teacher at the school summarized this burden in the following way:

“Women suffered more because even when somebody’s sick, if the husband is sick he is being taken care of by the wife. If the children are sick they are being taken care of by the woman. So you find out that they suffered more and they died more.”–>50 year old, male, Teacher

There were also geographic disparities, when comparing the experiences of people in rural and urban areas of the country. Participants shared their beliefs that fewer people living in urban areas died from Ebola but there were more instances of hunger in those areas, while there were more deaths due to Ebola in the rural areas (locally referred to as “the provinces”) but less hunger due to access to food crops.

#### Compromised culture

The Ebola epidemic impacted daily life in many ways; however, participants shared that one of the more challenging impacts of the epidemic was how it affected their ability to partake in important traditions with one another, particularly funeral and burial rites. A healthcare professional describes how her cultural traditions were compromised:

“You can’t expect my loved one to die and tell me I should not hold that person. As we are Africans, when someone died we feel sorry for that person, we sympathize with that person. But during Ebola, when someone died, they would tell you not to go to that home. Even when we have some security surrounding the entire building, you can’t go in there. There are so many things.”– 29 year old, female, Nurse

During the Ebola epidemic, individuals were unable to practice customary burial rites for their loved ones, whether they died of Ebola or other causes. Participants shared that this was very traumatic for families. One teacher noted:

“We lost our culture. People that could be buried in a respectful way, they just bury them like that. It was really sorrowful for us. It was not easy at that time”– 27 year old, male, Teacher

Participants also described how community members sometimes retaliated against the burial team when they were removing their loved ones’ bodies from the home. For instance, one stated:

“And it sometimes caused riots … Yes, among family members when this burial team, when they come to collect the corpse, some family members riot.”– 32 year old, male, Teacher

In addition to compromised funeral traditions, participants shared that they were unable to celebrate holidays and other customs. One grade six student noted, “*On the holidays we go to our village and celebrate our holidays*, *but because of Ebola we are unable to go there*.” Even typically happy traditions such as weddings could no longer be celebrated due to the inability of communities to gather together.

“When Ebola was here, marriages were postponed. Because in our local setting, whenever there is a marriage you have a reception and dance with each other and because of this transmission that one was shut up. There were no marriages.”– 53 year old, female, Nurse

#### Managing grief

Ebola has a high case fatality rate, and estimates suggest that almost 4,000 deaths can be attributed to the virus in Sierra Leone during the 2014–2016 epidemic [1]. In all of the interviews and focus groups we conducted, participants shared that they had lost community members and immediate family members during the Ebola epidemic. They expressed their understanding that traditional funeral practices were forbidden due to fears that they would lead to Ebola transmission; however, compromising funeral traditions also impacted how individuals could grieve for the loss of their loved ones. Even when families held small funeral services, people were hesitant about attending.

“There is no sympathy. Someone died, they want to attend the funeral? No. Because you are afraid. You don’t know what killed that person. You are afraid to attend the funeral. So you just decide, you just cry for the person, you just feel bad, but you will not go there. Except the close family members …”– 53 year old, female, Nurse

Compounding their grief, participants described that they did not know where their loved ones were buried, as individuals suspected of having Ebola were taken away in ambulances and sometimes never heard from again. Participants described the trauma and grief that they continue to feel over the loss of their spouses. One participant shared:

“They ask you out, take the body away from you, spray the house for any remaining virus, and then you will not be visiting them. And that happened to me. Up until now, I have never seen my wife again.”–>50 year old, male, Teacher

Another community member also shared her grief due to a similar situation:

“My husband was sick, and then even when he died, he finally died, I was going to go to see him, they forced me, they take him away from me, so I couldn’t do anything. And up until now, I’m still suffering because that space is still empty so I have to manage.”– 47 year old, female, Stonebreaker

Not all of the participants we interviewed had lost immediate family members during the Ebola epidemic; however, they did lose colleagues, friends, and members of their community. They described the grief for their community in the following passage:

“We cried a lot because seeing a colleague dying, seeing a member of your family dying, seeing a friend dying, seeing a relative dying, and you can’t do anything. You can’t touch them, you can’t bury them, you know it was terrible.”– 53 year old, female, Nurse

### Confusion and distrust

Fear, confusion, and misperceptions about Ebola were common during the Ebola epidemic. Additionally, nearly all participants expressed their lack of trust in the government and healthcare authorities’ response efforts during the Ebola epidemic. Because feelings of distrust and fear of healthcare services were so strong, many individuals did not seek these services even when they were needed, and this may have exacerbated the Ebola epidemic in Sierra Leone.

#### Belief that Ebola was man-made

Lack of communication from government officials and health workers created confusion about Ebola. Although most people learned about Ebola through the radio, many people, including healthcare workers, initially ignored the information.

“We just heard it’s a disease outbreak in some of the African countries-Ebola-that killed thousands of people, but … we never experienced it, so we just ignored it. We didn’t take it seriously.”– 29 year old, female, Nurse

Additionally, many people were suspicious that the disease was connected to an upcoming election. This led to widespread distrust toward the government with people speculating, *“When Ebola started*, *people were- they just thought that the government wanted to make money*. *Some of us did not believe*.*”– 29 year old*, *female*, *Nurse*

#### Misunderstanding Ebola transmission

Many participants shared that there was confusion about Ebola transmission during the epidemic, and indicated that people primarily misunderstood how they could contract the disease. Additionally, participants were still confused about Ebola transmission at the time of our interviews. One participant in the men’s focus group commented:

“I think that Ebola is like an airborne disease. I think Ebola is like airborne disease, right? Like TB, you know. Because of the air we breathe or whatever, then we can get Ebola.”– 42 year old, Construction Worker

In addition to not understanding Ebola transmission, some Sierra Leoneans believed that Ebola was a “curse” and not a disease. A healthcare professional relayed what she heard people say about the origins of Ebola in one village:

“What they say is that that they stole something from the neighbor village and they placed a curse on them. So that is their idea, they are going with that. It’s not Ebola. They even advise them not to eat bush meat, because at that time we don’t know, but we say bush meat can cause Ebola.”– 29 year old, female, Nurse

#### Fear of government services and healthcare

During the Ebola epidemic, people were afraid that if they reported their symptoms to health authorities, they would be sent to the health center and never see their families again. For instance, one participant stated:

“*And even if you are sick*, *you can’t go to the hospital*. *Because you are afraid*. *You think*, *when I go there*, *I will die*.*”–>50 year old*, *male*, *Teacher*

This led to a fear of government and healthcare services throughout the country. Even when healthcare services were used, participants expressed fear of the outcome of these services.

“Because the feeling was that no sooner you enter the ambulance, and you are taken to the hospital, well about call it 80% chance you would die.”–>50 year old, male, Teacher

There was also reluctance to alert the authorities about potential Ebola cases, as homes with an Ebola patient were quarantined for a period of 21 days after the patient was taken away [22].

“They invite the team- the burial team, they will come and take the specimen. After they take the specimen, they will know if the person die of Ebola or not. If the person die of Ebola, that particular household is going to be quarantined.”– 29 year old, female, Nurse

Additionally, due to the similarities between the symptoms of Ebola and malaria, people feared they would receive an incorrect diagnosis of Ebola. This led to an underreporting of illness and underutilization of healthcare facilities during the Ebola epidemic. One member of the school personnel stated:

“… and so people decided ‘well, let me die in my room.’ Because most people when they go [to the hospital], they will never come again.”–>50 year old, male, Teacher.

Participants also shared widespread reluctance to report their symptoms because they believed there was no purpose in seeking healthcare services since Ebola cannot be cured. Therefore, many ill individuals remained in their homes because they preferred to die at home than at the hospital. However, this put their families and caretakers at risk and contributed to Ebola transmission between family members.

"Well, again at first, we are taught that when you catch Ebola, you won’t be cured … So, with that people will say, ’Well, if I have Ebola I don’t see the reason why I should go to the doctor.’ So, they just decide to run away by themselves and hide in place. And at the end of it, they die there. And the family that is helping them and contacting them also die."– 32 year old, male, Teacher

Participants also expressed widespread fear and concerns about the methods officials used to prevent Ebola transmission. In particular, people were concerned about the chemical spray that officials used in their homes, reporting that they believed the prevention methods could have contributed to other health problems.

"When one person sick, he will report that feeling malaria, and all of us stay at this house. When they come, they will spray, and take everybody out, and spray, maybe one week, and after one week when you get inside that house all of us would die because of the chemical is too strong!"– 36 year old, female, Baker

In addition to concern about the chemicals such as chlorine that were used to treat the homes of suspected Ebola cases, community members also did not feel that the officials relayed proper instructions on how to stay safe during and after the spraying.

"When they first started spraying homes with chlorine, they did not tell people to wait three days before re-entering their homes. Like they are spraying a room, like when somebody has died, they will go test the person, the family, the whole family in there. Then after the spray they will say ’Ok this one is Ebola free’ but when they spray that area of the room, there is no ventilation and they will not tell the people to go out … So when you don’t go outside the people died. Maybe the whole family will die."– 33 year old, female, currently unemployed, previously a Home Health Educator

#### Trust of NGOs more than government

Although there was widespread distrust of the government, Sierra Leoneans trusted the work of NGOs and their services during the Ebola epidemic. NGOs provided a variety of services to families during that time, including food and water provisions and healthcare. Although the rations were less than their normal dietary intake, they eased the challenges many families endured during that time.

“No sooner the ambulance picked up the body, the NGOs- one of them will come- and see the number of people in that particular family. Maybe other ones will come and supply the food, the other one will give you water to drink, the other one will give you charcoal to cook. I mean, just like that. If you are fortunate on that day, if you are not fortunate then you have to, you have to bear it. But however if you are within the city, within three days you must get a little help. Although it is not enough. Because if you used to eat, call it four cups of rice and they say now you have to eat two, well you have to manage with two, because you have no alternative.”– 47 year old, female, Stonebreaker

### Moving forward after the epidemic

After the Ebola epidemic ended, concerns about transmission subsided over time; however, changes at the individual, interpersonal, and community level persisted after Sierra Leone was declared Ebola free on March 17, 2016. After the Ebola epidemic, participants shared that there was ongoing fear and stigma, and that they struggled with the loss of their loved ones, financial challenges, and the need to care for displaced family members.

#### Ongoing fear and stigma

Immediately after the end of the epidemic, community members continued to be fearful about Ebola transmission. Although Sierra Leone had been declared Ebola free, it was difficult for people to interact with one another like they had before the Ebola epidemic.

“I was afraid to go back to work. Because everybody is afraid of everybody. If you ask me to do a job for you, I am even afraid to come to your house. I’m even afraid to touch your children in your house. You give me water I am even afraid to drink water from your cup. Because I don’t know. Because it was very fearful.”– 42 year old, male, Construction Worker

Following the official end of the epidemic, fear and uncertainty still permeated the community. Ebola survivors were stigmatized by their fellow community members because they continued to be afraid of Ebola transmission.

“They are stigmatized … The President go to the village and announced that anyone that survived of Ebola that is being stigmatized- anyone that is stigmatizing that person should be taken to the police station. Because we are all one, that is what he told all the nation- that we are all one. We should not be stigmatizing. It can be to you, it can be to me. If someone survived, people think the best thing is we should avoid the person. Because of that, the president said that if you stigmatize someone, you are taken to the police, so it became lesser. So now we can interact with them, we can talk to them, yes.”– 29 year old, female, Nurse

Children were also stigmatizing survivors following their return to school as described by the fourth grade teacher.

“You know, kids are now using words against those that were affected. The stigma makes them to put far away from us, but we do encourage them to come to us because it was-just like it is meant to encourage them not to separate themselves from us. We are one. But that’s it- the stigma is a great thing that affected us up to now.”– 27 year old, male, Teacher

Teachers also expressed that they were initially fearful of putting themselves at risk when they returned to school after Sierra Leone was declared Ebola-free. However, this fear eventually subsided as classes continued and a sense of normalcy returned.

“For us in the lower classes during that time, they said nobody touch hands, but when we came to school, there was no boundary between us and the pupils. We rub our skins together, so it was really a risk for us as teachers.”– 44 year old, female, Teacher

#### Impact of losing loved ones

Following the fear of Ebola transmission, participants shared that they were more aware about hygiene and proper sanitation. However, a major challenge after the epidemic was managing how losing loved ones had impacted their daily life. For many people, the loss of family members was a poignant reminder of the epidemic and a difficult hurdle to overcome. Orphaned children struggled with the loss of their parents and had limited access to education. Additionally, some girls who were unable to continue their studies during the epidemic became pregnant.

“The effect of Ebola on Sierra Leone was great. Especially on the children who lost their parents. Because when you look at them- the majority of them are suffering. Because of the economic status of our country, you know, these children are not cared for. Most of them have dropped out of school, most of the girls have become pregnant, they are not going to school anymore. The boys are on the street …. so the lasting effect is there.”– 33 year old, female, currently unemployed, previously a Home Health Educator

#### Financial challenges

An unforeseen consequence of the Ebola epidemic was its impact on the economy of Sierra Leone. Even after Sierra Leone was declared Ebola-free, participants shared they were struggling financially. During the epidemic, food was scarce due to the reduction of imports into the country and the inability of commercial farmers and food manufacturers to continue working under government-imposed restrictions. The cost of food increased drastically as it became unavailable and community members had to change their eating habits due to limited accessibility and high food costs after the Ebola epidemic. One member of the school personnel explained the ongoing struggle to purchase food in the following passage:

“Things are becoming very hard, because, I can remember, for me, I used to buy a bag of rice for just 120, that was the baseline. And now it costs me 160 thousands … I buy sometimes certain rice that is very nice, very fine, and so a lot I buy half of that one for breakfast, right? But now I cannot do it, so instead I buy bread. And even at that is still costly. Because the bread we used to buy for 500 now is 1000, the one we used to buy for 1000 is 2000.”–>50 year old, male, Teacher

#### Caring for displaced family

Additional challenges after the Ebola epidemic included caring for family members who had lost their loved ones and opening their homes to children who were orphaned, which was a strain on their already limited finances.

"The cost of living. You know- too high. Because when you compare with before Ebola, you just stay with the children, but then after Ebola maybe your relative will lose a father and a mother, so all the family comes here. And so it’s not easy for us because the family is increased." - 33 year old, female, currently unemployed, previously a Home Health Educator

#### Continuing distrust of the government

After the epidemic ended, distrust of the government continued. Participants expressed knowledge that money was being donated from other countries and aid organizations to assist with Ebola recovery; however, it did not seem to them that the money was used for its intended purpose.

“People didn’t trust the government because there are rumors that payments are coming from everywhere. Money is sent for us that the facilities will be treated, but we did not see it … They didn’t use it on us, they only use it for their own interest, forgetting about the lower people.”– 27 year old, male, Teacher

Other community members felt that the government should financially assist victims of the epidemic, but that help was not forthcoming.

“It’s just a fact …..our government should pay victims, should help orphans, but on the whole, we are not seeing that … That mistrust is there ….We heard from other countries, like we were after Ebola has happened, like in Guinea what they did for their losses, but for us really I don’t know what is happening.”– 53 year old, female, Nurse

These feelings of fear, distrust, and frustration with government support were consistent across the adult focus groups and interviews.

## Discussion

To the best of our knowledge, our study is the first to examine the lived experiences of community members, including children, during and after the 2014–2016 Ebola epidemic in Sierra Leone. Additionally, our grounded theory methodology elucidates the unique lived experiences of community members, by drawing from, reassembling, and rendering subjects’ experiences during the Ebola epidemic [18].

Due to heightened fear, confusion, and loss during the epidemic, participants shared more about their experiences during that time; however, many challenges persisted after the Ebola epidemic. Additionally, our study focused on individuals who had experienced Ebola in their immediate families and in the surrounding communities, rather than solely focusing on the experiences of Ebola survivors, which have been reported elsewhere [23,24]. Findings from our qualitative interviews and focus groups reveal the suffering and daily challenges that community members experienced during the epidemic, the distrust of the government health system’s response to Ebola, and the residual effects of Ebola that persisted at the community level. It is important to note that the experiences described in our participants’ vignettes are likely unique to Sierra Leoneans with similar residences and socioeconomic characteristics. Moreover, these experiences were likely shaped by the history of the Civil War in Sierra Leone, as well as an inadequate health care system and disparities in resources that were shaped by colonial and postcolonial oppression. Additional studies are needed to examine the longevity of the epidemic’s impacts, including whether they persisted in Sierra Leone prior to and during the COVID-19 pandemic. Indeed, pre-existing structural conditions coupled with ongoing challenges after a recent public health emergency could influence how a country and its communities handle another emerging infectious disease.

Similar to other studies that examined experiences during the Ebola epidemic in Sierra Leone [10,12,25–28], we found that confusion about Ebola transmission and distrust of government healthcare services were common. Participants shared that they were fearful of Ebola response efforts, particularly the ambulance services and healthcare settings. Participants shared their concerns that the chlorine spray the government used to sanitize ambulances and homes of suspected Ebola cases was toxic and potentially lethal. These concerns echo those that have been reported in other studies, which found widespread beliefs that the chlorine spray the government used during the epidemic was harmful [10,25,26]. Participants also shared how their culture was compromised, particularly when they were unable to bury their loved ones according to their cultural and traditional practices. Barriers to safe burial acceptance during the Ebola epidemic have been reported in other studies [26,29]; therefore, future research should explore ways to improve community acceptance of safe burial practices as well as their trust in the government’s healthcare system.

Unlike other studies that mainly focused on experiences during the Ebola epidemic, participants of our study also shared their perspectives on the long-term impact that Ebola had on their community. For children, the loss of parents was particularly traumatic. Teenage pregnancy was associated with parent absence in conjunction with temporary school closures during the epidemic. Children who lost parents also faced stigma. This finding reflects that of Denis-Ramirez et al. who found children orphaned by Ebola were stigmatized by their peers and communities [30]. Many participants also shared that they continued to struggle with the loss of their loved ones. Experiences of anxiety or depression were not salient themes revealed in our study; however, a recent study using a national sample of Sierra Leoneans found individuals with any level of Ebola experience were more likely to report symptoms of anxiety, depression, and post-traumatic stress disorder (PTSD) [31]. Sierra Leoneans have been described as having a “culture of resiliency” and this could influence low observations of clinical anxiety and depression after the Ebola epidemic [31,32]. This observation could explain why the proportion of individuals who exhibited clinical levels of anxiety and depression in the Sierra Leonean study by Jalloh et al. (2018) was lower than expected [31], and why participants in our interviews and focus groups did not share feelings of anxiety or depression. Another possibility is non-verbal cues of anxiety and depression may differ between cultures, and our interviewers, who come from Western backgrounds, may not have observed these cues during the focus groups and interview process. Financial challenges also persisted, largely related to shifts in food costs after the epidemic. Participants also shared that providing for children and adults who lost loved ones during Ebola was a stress on the already challenging financial situation in the community. Additional future research from a life course perspective is warranted to examine whether or not these experiences could have long-term impacts on health, including the health of future generations.

Our study is limited by the generalizability of our findings to other communities within and outside of Sierra Leone. Because the Calaba Town community is approximately 20 km southeast of Freetown, the experiences participants shared may be unique to communities outside of the city limits but not in the more rural provinces. However, because participants shared similar responses between the two years of interviews and because we reached data saturation, we are confident that the experiences participants shared are similar to those of other individuals in the Calaba Town community. Although we did not interview individuals who had survived Ebola, the participants had experienced Ebola in their families and among their neighbors. Another possible limitation of our study is related to the selection process. Participants were selected via purposive sampling. That is, personnel from the school and members of the small community with whom members of the research team had rapport were invited to participate. While this process could have potentially led to selection bias, it also was the foundation for building trust and openness. Alternatively, because of our close relationship with the community, social desirability bias could have influenced some participants’ responses. Finally, recall bias is a possibility for questions about experiences during the Ebola epidemic; however, because the study was conducted shortly after the epidemic ended, we believe participants’ recollections were not impacted by a time lapse between the epidemic and the focus group or interview. Despite these limitations, we believe that our study adds to the body of literature on the community effects of the Ebola epidemic.

The recent development of an Ebola vaccine has been promising [33]. However, the catastrophic consequences of infectious diseases like Ebola require public health practitioners to continually focus on improving quality of life for individuals both during and after epidemics. While the government of Sierra Leone acted quickly in response to COVID-19 [34], we do not yet know how experiences during the Ebola epidemic, including distrust, fear, loss, and compromised culture, might influence people’s responses to government efforts during the pandemic. As such, findings from this qualitative study can inform healthcare officials, NGOs, and the general public on public health needs during and after epidemics, which extend beyond preventing disease transmission and providing immediate health care services. Additionally, it is critically important to ensure the public’s trust in the government’s response efforts and strengthen the healthcare system.

## Conclusion

The 2014–2016 Ebola epidemic in Sierra Leone was a devastating public health crisis. Although it is imperative that we identify effective response efforts to such contagious infectious diseases with high mortality, we must additionally understand the lived experiences of community members, including children, before and after such catastrophic epidemics. First-hand accounts from individuals who witnessed and experienced Ebola can help us better understand how epidemics due to infectious diseases with high mortality impact daily life, including their long-term consequences.

The findings of this qualitative study revealed that the Ebola epidemic was a traumatizing period with daily life challenges, as well as feelings of confusion and distrust toward the government health care system. However, many challenges continued after the Ebola epidemic. Ongoing fear and stigma, financial challenges, and the need to care for displaced family members continued to impact the Calaba Town community. These challenges should be explored further in other studies, particularly the long-term impact that devastating epidemics such as this one can have on communities. Research that focuses on the experiences and consequences of epidemics at the community level, as well as their impact on children, will be informative for future response efforts during widespread public health crises, especially infectious disease outbreaks. Given the subsequent epidemic of Ebola in the Democratic Republic of Congo in May of 2018 and the COVID-19 pandemic, there is an urgent need for additional research that examines how outbreaks such as these can impact daily life.
